# Thiopental and Phenytoin as Novel Ionophores for Potentiometric Determination of Lead (II) Ions

**DOI:** 10.3390/s90301860

**Published:** 2009-03-13

**Authors:** Nashwa M.H. Rizk, Samah S. Abbas, Salem M. Hamza, Yasser M. Abd EL-Karem

**Affiliations:** 1 Environmental Biotechnology Department, Genetic Engineering and Biotechnology Research Institute (GEBRI), Minufiya University, Sadat City, Egypt; 2 Pharmaceutical Analytical Chemistry Department, Faculty of Pharmacy, Cairo University, Egypt; E-mail: samah_abbas2005@yahoo.com; 3 Chemistry Department, Faculty of Sciences, Minufiya University, Egypt E-mail: salem.hamza@y.com; yasser_karem_1981@yahoo.com

**Keywords:** Lead (II), Thiopental, Phenytoin, Galena rocks, Solder alloys

## Abstract

Two novel polymeric membrane sensors for the analysis of Pb(II) have been developed based on two therapeutic drugs, thiopental (TP) and phenytoin (PT) as two new ionophores and potassium tetrakis(*p*-chlorophenyl) borate (KT*p*ClPB) as a lipophilic additive, in plasticized PVC membranes. The sensors show a Nernstian response for Pb(II) ions over the wide concentration ranges of 1×10^−2^ – 7×10^−6^ M and 1×10^−2^ – 8×10^−6^ M for the sensors based on thiopental and phenytoin, respectively. The proposed sensors have a fast response time and can be used for more than nine weeks without any considerable divergence in potentials. The sensors exhibit comparatively good selectivity with respect to alkaline, alkaline earth and some transition and heavy metal ions. They were employed for direct determination of lead in solder alloys and in galena rocks with a good agreement with the obtained results by atomic absorption spectroscopy.

## 1. Introduction

Lead is an environmental pollutant that accumulates with toxic effects in blood, liver, kidney and in the central nervous system of exposed mammals. The neurotoxicity of lead is of special interest, since cognitive and motor deficits in children have been associated with low levels of lead exposure [1,2]. The nephrotoxicity of lead has also been extensively studied, since the initial accumulation of absorbed lead occurs primarily in the kidney, where it may cause proximal tubular dysfunction after acute exposure, or irreversible nephropathy that may lead to renal failure after chronic exposure [1].

Assessment of accumulation, deficiency and concentration of lead levels require sensitive, reproducible and accurate analytical techniques. Methods in current use for lead quantification include stripping voltammetric-potentiometric electrodes [3,4], hydride generation-ICP OES [5], isotope dilution inductively coupled plasma mass spectrometry [6], electrothermal atomization laser excited atomic fluorescence [7], atomic absorption spectrometry [8–10], voltammetry [11,12], spectrophotometry [13], coulometry [14], and potentiometric methods [15–19].

Bis(2-hydroxyacetophenone)ethylenediimine [20], *N,N′-*bis(2-hydroxy-1-napthalene)-2,6-pyridinamine [21], *N,N’*-dibenzyl-1,4,10,13-tetraoxa-7,16-diazacyclooctadecane [22], piroxicam [23], dioxamides [24,25], 9,10-anthraquinone derivatives [26], derivatized tetrapyrazole and calix[4]arene receptors [27], monobis- and pentakis-derivatives of *p-tert*-butylcalixarene [28,29], calixarene phosphine oxide derivatives [30], 4-*tert*-butylcalix[4]arene [31], calix[4]arene amide [32], some fatty acids [33], 3,6-dioxaoctanedithioamide [34], a crown ether [35], a thiacrown derivative [36], a substituted diaza-18-crown-6 [37], tetrabenzy1 pyrophosphate and diphenylphosphinic anhydride [38], a Schiff's base complex [39] and methoxy substituted arylenevinylene derivatives [40] are used as electroactive materials in lead (II) selective membrane sensors. Advantages and limitations of some of these sensors are discussed in this work.

**Figure 1.**
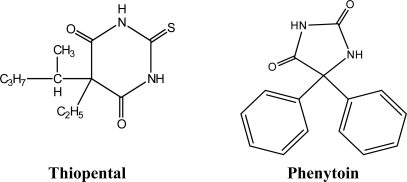
Chemical structure of lead (II) ionophores.

Thiopental (also called pentothal, Figure 1), a thiobarbiturate, is used for the induction of anesthesia prior to the use of other general anesthetic agents and for short surgical, diagnostic, or therapeutic procedures associated with minimal painful stimuli. It is an ultrashort-acting depressant of the central nervous system which induces hypnosis and anesthesia [41]. This drug is also used in the induction phase of general anesthesia, because of how quickly it takes effect. It usually takes less than 30 seconds for the drug to reach the brain and cause unconsciousness. It is not used for prolonged anaesthesia due to the excessive amount of time it takes for the patient to regain consciousness after induction. Instead, inhaled agents are used so that moments after the inhaled agent is removed, the patient regains consciousness [42].

Phenytoin (5,5-diphenylimidazolidine-2,4-dione, Figure 1) is a commonly used antiepileptic. Barbiturates are widely used as sedative hypnotic drugs in a wide variety of conditions and are also employed to produce anesthesia [43]. Phenytoin is one of the cyclic ureides which related in structure to the barbiturates. It was reported to be the least hypnotic, most strongly anticonvulsant and most effective against grand mal [43]. Phenytoin is extensively metabolized in the liver to 5-(*p*-hydroxy-phenyl)-5-phenylhydantoin and between 60 and 70% of the administered dose is excreted as free or as a glucuronide conjugate of 5-(*p*-hydroxyphenyl)-5-phenylhydantoin. Minor metabolises include 5-(3-hydroxyphenyl)-5-phenylhydantoin, 3,4-dihydro-3,4-dihydroxyphenytoin, catechol and 3-*O*-methyl-catechol. The plasma half-life varies considerably within the approximate range of 7–60 h and is dose-dependent [42].

In this work, new lead sensors incorporating the two ionophores thiopental and phenytoin were used as electroactive materials in a plasticized PVC matrix. These sensors exhibit significantly high sensitivity, stability, selectivity for Pb(II) ions over many common ions and are successfully used for determining lead (II) ions in various matrices.

## 2. Experimental

### 2.1. Equipment

Potentiometric measurements were made at 25±1°C with an Orion digital ion-analyser (model, 420A) using the proposed sensor with thiopental and phenytoin as ionophores dispersed in PVC matrix and plasticizer as solvent mediator. Lead membranes were used in conjunction with an Orion, Ag/AgCl double-junction reference electrode containing 10% (w/v) KNO**_3_** solution in the outer compartment. Adjustment of the pH was made with an Orion 91-20 combination glass electrode. The cell assembly for the measurement of potential is Ag/AgCl/KCl (0.1 M), KNO**_3_** 0.1 M/sample test solution//sensor membrane//internal filling solution /AgCl/Ag. Atomic absorption spectrometric measurements of Pb**^2+^** were made with Perkin–Elmer spectrometer (model 3100) using the recommended optimum conditions [44].

### 2.2. Reagents

All chemicals were of analytical-reagent grade, and bidistilled deionized water was used throughout. Thiopental and phenytoin were obtained from Sigma Chemical Co. (St. Louis, MO., USA). *o*-Nitro-phenyloctyl ether (*o*-NPOE), dioctylphthalate (DOP), and tetrahydrofuran (THF) were obtained from Aldrich Chemical Co. (Milwaukee, Wisconsin, USA). Aqueous 1×10^−^**^2^**–1×10^−7^ M lead solutions were freshly prepared by accurate dilutions of a standard 0.1 M stock lead acetate solution by using 0.01 M acetate buffer of pH 5.5.

### 2.3. Sensor Construction

The general procedure for preparation of the PVC membrane is similar to that previously described [16]. The membrane was prepared by mixing ∼/2 mg portions of thiopental or phenytoin ionophores in a glass Petri dish (5 cm diameter) with ∼/132 mg of *o*-NPOE and ∼/66 mg of PVC. The mixture was dissolved in 5 mL of THF. The Petri dish was covered, and left to stand overnight to allow slow evaporation of the solvent at room temperature. A master PVC membrane (0.1 mm thickness) was obtained. The internal reference solution was prepared by mixing equal volumes of 0.01 M KCl and Pb(CH_3_COO)_2_.

### 2.4. Calibration of Lead Membrane Sensors

The membrane sensors were calibrated by immersion in a 1×10^−7^–1×10^−2^ M lead acetate solution with 0.01 M acetate buffer of pH 5.5. An aliquot (1.0 mL) of each solution was transferred into a 50 mL beaker containing a 0.01 M acetate buffer solution of pH 5.5 (9 mL). Proposed sensors were immersed and allowed to equilibrate with constant stirring in conjunction with an Orion reference electrode. The sensors were washed with bidistilled deionized water between measurements. The electrode potential was recorded as a function of the lead concentration. The obtained calibration plot was used for subsequent measurements of unknown lead concentration under the same conditions.

### 2.5. Sensor Selectivity

The potentiometric selectivity coefficient ( $K_{\mathrm{pb}(\mathrm{II}),B}^{\mathrm{Pot}}$) of the lead sensors was measured by the separate solutions method [45,46]. Different concentrations of each interferent and lead solutions (1×10^−7^–1×10^−2^ M) were prepared in 0.01 M acetate buffer of pH 5.5; the potentials were measured and plotted against concentration. The selectivity coefficients were calculated using the following equation:

$$K_{\mathrm{pb}(\mathrm{II}),B}^{\mathrm{Pot}}=a_{\text{pb}(\text{II})}/{\left(a_{B}\right)}^{Z_{\text{Pb}(\text{II})}/Z_{B}}$$

where a_pb(II)_ and a_B_ are the activities of the primary ion Pb**^2+^** and interfering ion respectively; Z_Pb(II)_ and Z_B_ are the charges of Pb**^2+^** and interfering ions respectively.

### 2.6. Analytical Applications

#### 2.6.1. Determination of Lead in Solder Alloys

About 1.0 g of sample was added to concentrated nitric acid (10 mL) until the sample was dissolved, then the solution was diluted by the addition of acetate buffer (pH 5.5, 40 mL). The pH was adjusted at pH 5.5 using acetic acid and NaOH. It was then filtered to separate tin. The concentration of lead (II) ions was determined by standard addition methods. Besides, lead in sample solution was determined by using atomic absorption spectrometry to compare the results with the potentiometric method.

#### 2.6.2. Determination of Lead in Rocks

A well ground portion of galena, a lead ore rock (about 2 g) was transferred to a porcelain crucible and digested according to the standard method [47] to convert lead sulfide into lead (II) ions. K_2_S_2_O_7_ (10 g) was added to the rock powder, the components were mixed and the mixture was heated for 30 min on a hot plate. After cooling, a portion of (1:1) aqueous HNO_3_ (60 mL) was added and the crucible was heated on a hot-water bath until the melt disintegrated. The crucible was intermittently shaken, the melt was crushed with a glass rod and the crucible was removed from the water bath. The sample was filtered off, washed with (1:1) HNO**_3_** (10 mL) followed by hot distilled water (50 mL) and the contents were transferred to a 100-mL volumetric flask. The solution was adjusted to the mark with deionized water and shaken. An aliquot of the sample (1.0 mL) was transferred into a 50 mL beaker followed by acetate buffer solution of pH 5.5 (1 mL). The solution was diluted with deionized distilled water to a total volume of ∼/10 mL and adjusted to pH 5.5 using acetic acid and NaOH. The lead (II) membrane sensors and a double junction Ag/AgCl reference electrode were immersed in the solution and the concentration of Pb(II) was measured by direct potentiometry. Lead in sample solution was determined by using atomic absorption spectrometry to compare the results with the potentiometric method.

## 3. Results and Discussion

### 3.1. Characteristics of the Sensors

The lead PVC membrane sensors based on the ionophores TP (Sensor 1) and PT (Sensor 2) (see Table 1) with the composition: ∼1 wt.% ionophore, 33 wt.% PVC and 66 wt.% plasticizer (*o*-NPOE) exhibit linear responses to Pb^2+^ ions within the concentration range of 1×10^−2^–9×10^−6^ M and 1×10^−2^–1×10^−5^ M with lower detection limits of 7×10^−6^ M and 6.5×10^−6^ M, respectively. In the presence of ∼ 0.05 wt.% of KT*p*ClPB as a membrane additive, and 1 wt.% ionophore, 33 wt.% PVC and 66 wt.% plasticizer (*o*-NPOE) the detection limit, linear range and calibration slope [48] are improved. The lower detection limits are 5×10^−6^ M and 4.5×10^−6^ M and the linear ranges are 1×10^−2^ – 7×10^−6^ M and 1×10^−2^ – 8×10^−6^ M for membrane sensors incorporating ionophores TP and PT, respectively. Both sensors exhibit near-Nernstian slope of 31.5 mV per decade (correlation coefficient 0.998), and 30.5 mV per decade (correlation coefficient 0.999), respectively (n = 6).

Lead PVC matrix membrane sensors incorporating ionophore (PT) and different plasticizer having various dielectric constants (e.g. DBS, DOP and *o*-NPOE) were prepared and tested. Membrane sensors based on the PT ionophore (Sensors 8, 6, 2) plasticized with DBS (***ε*** = 4), DOP (***ε*** = 7), and *o*-NPOE (***ε*** = 24) show calibration slops of 21.6, 23.6 and 27.3 mV per decade with linear ranges of 5×10^−3^−1×10^−4^, 5×10^−3^–6×10^−5^ and 1×10^−2^−1×10^−5^ and lower detection limits of 8×10^−5^, 1×10^−5^ and 6.5×10^−6^ M, respectively. With membrane sensors based on the TP ionophore (Sensors 7, 5, 1), calibration slopes of 22.5, 25 and 28.5 mV per decade with linear ranges of 5×10^−3^−8.5×10^−5^, 5×10^−3^−1×10^−5^ and 1×10^−2^–9×10^−6^ M are obtained with DBS, DOP and *o*-NPOE membrane plasticizers, respectively. It can be seen that sensor based on the PT ionophore in a DBS plasticized membrane (Sensor 8) shows lower slope and detection limit, while the sensor based on the TP ionophore in an *o*-NPOE plasticized membrane (Sensor 1) shows higher slope and detection limit. This may be due to the highest dielectric constant of *o*-NPOE than DBS. Table 1 shows the optimization of membranes ingredients of lead sensors based on ionophores (TP) and (PT) with different plasticizers in absence and presence of KT*p*ClPB as a membrane additive.

Table 2 shows the performance characteristics of the lead membrane sensors based on the ionophores TP and PT with *o*-NPOE in presence of KT*p*ClPB. It is well known that the addition of lipophilic ionic sites to an ion-membrane sensor based on a neutral ionophore is necessary to reduce the interference of lipophilic counter-ions, for fast response time, to decrease the electrical resistance of the membrane and to improve the selectivity [46,49]. Calibration slopes of electrode with 0.05 wt% of potassium tetrakis(*p*-chlorophenyl)borate (KT*p*ClPB) showed that the electrode has the slope 31.5 mV per decade for TP and 30.5 mV per decade for PT. Also the detection limits were improved by using anionic additive (KT*p*ClPB) with the membrane matrix (Table 1). The robustness of an analytical method is a measure of its capacity to remain unaffected by a small but a deliberate variation in method parameters and provide an indication of its reliability during normal usage [50]. While the ruggedness of an analytical method is the degree of the reproducibility at test results obtained by the analysis of the same samples under a variety of conditions such as different laboratories, analysts and instruments [50]. The results obtained by using pH-meter (Orion 420A) were compared with those obtained using another model of pH-meter (Jenway 720). The obtained results are close and also reveal the validity of the method. The slight differences in lipophilicity, ring size and polarity of the two ionophores (see Figure 1) do not significantly affect the general electrochemical performances of the sensors (Table 2).

The suggested mechanism for the proposed lead (II) ionophores is as follows: the lead forms a stable five membered chelating ring with thiopental and phenytoin in a 1:2 lead (II) to drug ratio. The mechanism was proven by applying Job's method, which is an extremely versatile approach to the determination of reaction stoichiometries [51].

The dynamic response times of sensors based on the TP and PT ionophores with *o*-NPOE in the presence of KT*p*ClPB to reach ∼95% of equilibrium response are ∼20 s for both sensors. In general the TP and PT ionophore-based based sensors exhibit similar responses characteristics for Pb^2+^ ions. The response time of the concentrations from 1×10^−5^ to 1×10^−2^ varies between 15–20 s. The nearly identical response time on varying the metal ion concentration is probably due to the fast exchange kinetics of complexation–decomplexation of Pb^2+^ ion at the test solution/membrane interface.

The lifetime of the sensors was detected by measuring the slope of the potential versus lead ion concentration over the concentration range of 1×10^−5^–1×10^−2^ M each week over a period of nine weeks while the electrode was in continual use. The slope remained constant through the assessment period. However, a slight change in the response was found and corrected by reconditioning the electrode by soaking it in a 0.01 M solution of Pb(CH**_3_**COO)_2_ for 24 h. The sensors long lifetime are due to that, the ionophore is well soluble in the membrane matrix and the stable complex formation between lead ions and suggested ionophores.

### 3.2. Effect of pH and Foreign Ions

**Figure 2.**
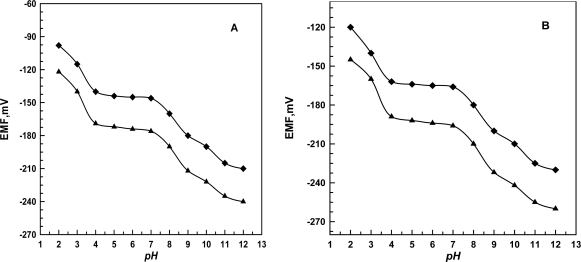
Effect of the pH on the potential responses of; (A) (PT) sensor with additive and (B) (TP) sensor with additive at (♦) 1.0×10^−2^ M and (▴) 1.0×10^−3^ M lead concentration.

A study of the potential-pH curves of Pb^2+^ membrane sensors based on TP and PT ionophores reveals that within the range 4.0–7.0, the potential did not vary by more than ±2 mV. At pH > 7, the emf values of both sensors sharply decrease due to the precipitation of Pb(OH)_2_ and/or formation of hydroxyl lead complexes and competition of OH^−^ ion with the ionophores for Pb^2+^ ions. At pH< 4, interferences from H^+^ ions are significant with subsequent increasing in the potential response. All subsequent potentiometric measurements of Pb^2+^ ions were made in 10^−2^ M acetate buffer background of pH 5.5 (Figure 2).

**Figure 3.**
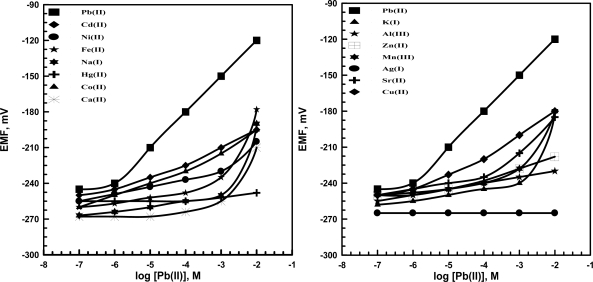
Potentiometric responses of lead membrane sensors based on (TP) with additive as ionophore toward several metal ions.

**Figure 4.**
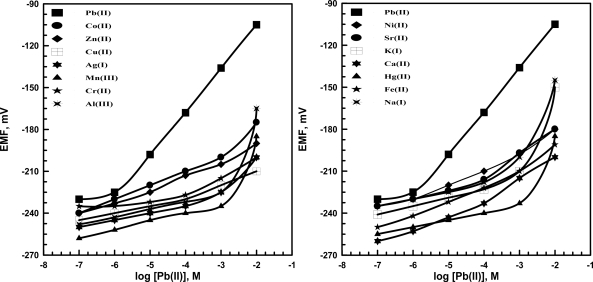
Potentiometric responses of lead membrane sensors based on (PT) with additive as ionophore toward several metal ions.

The potentiometric selectivity coefficients ( $K_{\mathrm{pb}(\mathrm{II}),B}^{\mathrm{Pot}}$) of lead sensors using KT*p*ClPB based on TP (Figure 3) and PT (Figure 4) (*o*-NPOE plasticizer) were determined using the separate solutions (SSM) method [45,46]. Another assembles of sensors were used for selectivity determinations rather than that used for pH measurements. Then different concentrations of each interferent and lead solutions (1×10^−7^–1×10^−2^ M) were prepared in 0.01 M acetate buffer of pH 5.5; the potentials were measured and plotted against concentration. The results reveal that high concentrations of most cations do not affect the selectivity of the sensors towards Pb^2+^ ions. In presence of KT*p*ClPB as anionic additive the selectivity of lead sensors were improved more than without additive. This means that the coordination of lead (II) ions by the ionophores can also be related to the presence or the absence of lipophilic anionic sites, which is usually added to such membranes.

The stability of complexes is governed by the pH of the metal solutions for Ca^2+^, Sr^2+^ and Mg^2+^; form stable complexes at pH 8–10. Fe^2+^ form stable complexes in acidic medium. Metals; Na^+^ and K^+^ can not form stable complexes, while Al^3+^ can react slowly and form weak complexes. Lead (II) ion can form stable complex over some other heavy metals [52]. The selectivity studies of lead (II) membrane sensors emphasis the superiority of the sensors towards lead (II) over many other metals.

Table 3 represents the selectivity coefficients of PT and TP based lead sensors with and without additives at 10^−3^ M of lead ions and interferent. Membrane sensor based on TP and in presence of additive exhibits slightly better selectivity for Pb^2+^ ions over metal cations compared to other sensors.

### 3.3. Comparison with Other Reported Lead Sensors

In Table 4, the response characteristics and the selectivity coefficients of the proposed sensors towards some potential interfering ions are compared with corresponding values previously reported for lead ion-selective membrane sensors based on a variety of different ionophores [16,22–24,33,36,39,40]. As can be seen, the linear range and the response time of the proposed sensors are superior to some of those reported for other lead ion-selective membrane sensors and the selectivity's behaviors are among the most selective Pb^2+^ ion sensor reported. The superiority of TP and PT ionophores over some of other reported ionophores for lead (II) is attributed to the high stability of their lead (II) complexes.

### 3.4. Direct Determination of Pb^2+^ Ions

Determination of Pb^2+^ ions using the TP or PT based ionophore lead sensors was validated according to the quality assurance standards [53]. Five batches (15 μg mL^−1^) were used (five determination each) for measuring accuracy, precision, linear range, lower limit of detection, repeatability (CV_w_) and between-day-variability (CV_b_).

### 3.5. Analytical Applications

#### 3.5.1 Determination of Lead in Solder Alloys

The sensors based on TP and PT were employed to determine the lead (II) concentration in real samples. Lead is used in a number of alloys, particularly low-melting alloys, such a solder, A commercial solder alloy purchased from Save (Vitoria, Spain), containing mainly lead and tin, was used in this study. Lead in the sample was put into solution by treatment with concentrated nitric acid to dissolve lead and to precipitate tin in acidic media; if concentrated hydrochloric acid was added, the tin would be re-dissolved. Since the aim of this analysis is to determine lead in solder alloys, it is better to remove any tin of the sample solution, In fact, better results, without any tin interference, were obtained when the sample treatment was only made with concentrated nitric acid. The obtained results in Table 5, which have been showed a lead percentages of 70.2 ± 0.5 (Sensor 3) and 69.8±0.4 in solder alloys. In order to show that potentiometric sensors are suitable to make this determination, the samples were analyzed by atomic absorption spectrometry [8]. The obtained result of lead percentage is 66.9±0.8. In this way, lead determination data by the potentiometric method are in good agreement regarding to precision (F) with the obtained results by atomic absorption spectroscopy. But we found significant difference between the proposed and reported [8] methods regarding accuracy (t-test), Table 5.

#### 3.5.2 Determination of Pb^2+^ in Galena Rocks

The lead content of some natural rocks (e.g. galena) was assessed. The rocks were dissolved in nitric acid and the lead (II) solution was measured by direct potentiometry using TP and PT ionophore based membrane sensors. The obtained results by potentiometric sensors in Table 6, which have been showed a lead percentages of 12.5 ± 0.6 (Sensor 3) and 12.3 ± 0.4 (Sensor 4) in galena rocks. Similar result (12.5 ± 0.8) was obtained using atomic absorption spectrometry [8]. As can be seen, we found from the statistical methods, data in good agreement with reference method regarding accuracy (t-test) and precision (F value).

### 3.6. Conclusions

The main advantage of the potentiometric sensor is its simplicity of preparation, short conditioning time, fast response time, Nernstian behavior and improved good selectivity. The membrane is long-lived and chemically stable. The sensor was successfully applied to the direct determination of Pb(II) in solder alloys and galena rocks.

## Figures and Tables

**Table 1.** Optimization of membrane ingredients.

**Sensor No.**	**Composition (mg)**				**Slope (mV decade^−1^)**	**Linear range (M)**	**Lower detection limit (M)**
	**Ionophore**	**PVC**	**Additive**	**plasticizer**			
1	2.0 (TP)	66.0	-	132.0 (*o*-NPOE)	28.5	1×10^−2^–9×10^−6^	7.0×10^−6^
2	2.1 (PT)	66.1	-	131.7 (*o*-NPOE)	27.3	1×10^−2^–1×10^−5^	6.5×10^−6^
3	1.9 (TP)	65.9	0.1 (KT*p*ClPB)	132.1 (*o*-NPOE)	31.5	1×10^−2^–7×10^−6^	5.0×10^−6^
4	2.0 (PT)	65.7	0.1 (KT*p*ClPB)	132.0 (*o*-NPOE)	30.5	1×10^−2^–8×10^−6^	4.5×10^−6^
5	1.8 (TP)	66.2	-	131.8 (DOP)	25.0	5×10^−3^–1×10^−5^	8.0×10^−6^
6	2.1 (PT)	66.0	-	132.2 (DOP)	23.6	5×10^−3^–6×10^−5^	1.0×10^−5^
7	2.0 (TP)	66.4	-	131.6 (DBS)	22.5	5×10^−3^–8.5×10^−5^	4.0×10^−5^
8	1.9 (PT)	66.1	-	132.4 (DBS)	21.6	5×10^−3^–1×10^−4^	8.0×10^−5^

**Table 2.** Potentiometric response characteristics of *o*-NPOE plasticized PVC membrane sensors with KT*p*ClPB.

**Parameter^a^**	**Sensor 3**	**Sensor 4**
Slope (mV decade^−1^)	31.5	30.5
Intercept (mV)	−330	−270
Correlation coefficient (r) (n=6)	0.998	0.999
Linear range (M)	1×10^−2^–7×10^−6^	1×10^−2^–8×10^−6^
Lower limit of detection (M)	5.0×10^−6^	4.5×10^−6^
Response time for 10^−3^ M (s)	∼20	∼20
Working pH range	4–7	4–7
Life span (week)	>9	>9
Accuracy (%)	99.3	99.0
Standard deviation (%)	0.7	0.6
Repeatability (CV_W_%)	0.8	0.7
between day variability (CV_b_%)	0.9	1.0
Robustness^b^	101.4 ± 1.7	102.0 ± 1.2
Ruggedness^c^	100.6 ± 1.5	101.7 ± 1.6

a Mean of six measurements.

b A small variation in method parameters were studied as pH of buffer.

c Comparing the results by those obtained by different sensors assemblies using Jenway 720 potentiometer.

**Table 3.** **S**electivity coefficient ( $K_{\mathrm{pb}(\mathrm{II}),B}^{\mathrm{Pot}}$) of Lead sensors based on PT and TP as ionophores at 10^−3^ M for both Pb^2+^ and interferent.

**Interfering ion^a^**	log $K_{\mathrm{pb}(\mathrm{II}),B}^{\mathrm{Pot}}$			
	**TP without additives**	**TP with additives**	**PT without additives**	**PT with additives**
Co^2+^	−2.06	−3.17	−2.01	−2.16
Hg^2+^	−1.17	−1.5	−1.07	−1.37
Ca^2+^	−2.77	−5.0	−2.12	−4.19
Sr^2+^	−2.17	−3.96	−2.4	−3.12
Zn^2+^	−2.31	−2.38	−2.27	−2.83
Cu^2+^	−1.06	−1.58	−1.01	−1.53
Mn^2+^	−2.17	−3.34	−3.14	−3.52
Ag^+^	−2.0	−2.52	−2.0	−2.69
Na^+^	−3.86	−5.36	−3.51	−3.67
K^+^	−3.1	−4.66	−3.3	−3.9
Fe^2+^	−2.52	−3.62	−2.67	−3.34
Al^3+^	−2.67	−3.0	−3.35	−4.33
Ni^2+^	−1.68	−2.34	−1.85	−2.06
Cd^2+^	−2.33	−2.37	−1.34	−2.03

a. Mean of five measurements

**Table 4.** General performance characteristics of some potentiometric lead membrane sensors.

**Ionophore**	**Linear Range (M)**	**Lower limit of detection (M)**	**Slope (mV/decade)**	**Interferent (M, Selectivity)**	**Ref.**
9,10-Anthraquinone derivatives	1×10^−6^ – 1×10^−2^	6.7×10^−7^	28.9	Zn^2+^,Cd^2+^	[22]
Methoxy substituted arylenevinylene derivatives	4.2×10^−4^ – 2.0×10 ^−2^	NR	33–36	Na^+^ −1.33, K^+^ −1.66, Mg^2+^ −1.3, Zn^2+^ −1.3, Cd^2+^ −1.28, Ca^2+^ −1.39, Cu^2+^−0.17, Ni^2+^−1.11	[40]
Dioxamide	1×10^−6^ – 8.4×10 ^−3^	NR	31.9	Hg^2+^ −1.6, Fe^2+^ −1.67, Cd^2+^−2.1	[24]
Thia crowm derivatives	1×10^−6^ – 8×10^−3^	8×10^−7^	29	Hg^2+^ −2.1	[36]
Piroxicam	1×10^−5^ – 1×10^−2^	4×10^−6^	30	UO^2+^ −0.43, Ag^+^ −1.2.K^+^ −1.29, Zn^2+^ −1.08, Mg^2+^−1.24	[23]
*N*,*N*’-bis(salicylidene)-2,6-pyridinediamine	NR	9.12×10^−7^	29.4	Na^+^ −2.5, K^+^ −2.2, Ag^+^ −2.2, Zn^2+^ −4.1, Co^2+^ −4.2, Mg^2+^ −4.9, Cu^2+^−2.7	[39]
Chiral 2,6-bis-pyridine-carboximide derivatives	9×10^−6^– 1×10^−2^	4.4×10^−6^	21.6	Li^+^ −3.4, Na^+^ −3.41, K^+^ −3.5, Ca^2+^ −1.45, Cu^2+^−1.06, Cd^2+^ −1.61, Ag^+^ −2.89, Hg^2+^ −1.00.	[16]
	5.8×10^−5^ – 1×10^−2^	1.8×10^−5^	33.1	Li^+^−3.83, Na^+^ −4.24, K^+^ −3.83, Ca^2+^ −2.14, Cu^2+^−2.03, Cd^2+^ −2.17, Ag^+^ −2.25, Hg^2+^ −2.10.	[16]
	4×10^−6^–1×10^−2^	2.1×10^−6^	25.0	Li^+^ −4.12, Na^+^ −3.70, K^+^ −4.11, Ca^2+^ −1.91, Cu^2+^−1.99, Cd^2+^ −1.94, Ag^+^ −2.89, Hg^2+^ −1.5.	[16]
Fatty acids	1×10^−6^–1×10^−2^	NR	29	Ag^+^ −0.9. K^+^−0.89, Na^+^−0.80	[33]
Thiopental (Sensor 3)	4.5×10^−6^ − 1×10^−2^	1×10^−6^	30.5	Na^+^−3.86, Zn^2+^ −2.38, Mn^2+^−2.17, Cd^2+^ −1.37, Ag^+^ −3.52, K^+^−4.66, Ca^2+^ −3.0, Cu^2+^−2.08	This work
Phenytoin (Sensor 4)	6.4×10^−6^– 1×10^−2^	1×10^−6^	31.5	Na^+^−3.67, Zn^2+^ −2.83, Mn^2+^−3.52, Cd^2+^ −1.34, Ag^+^ −3.0. K^+^ −4.19, Ca^2+^ −1.19, Cu^2+^−2.03	This work

NR, not reported

**Table 5.** Determination of lead in solder alloy by using potentiometric sensors and atomic absorption spectrometry (AAS) techniques.

**Sample**	**Lead content^a^ (mg/g)**		
	**Sensor 3**	**Sensor 4**	**AAS [8]**
1	70.2 mg ± 0.5 mg	69.7 mg ± 0.4 mg	66.7 mg ± 0.9 mg
2	70.0 mg ± 0.6 mg	69.9 mg ± 0.5 mg	66.9 mg ± 0.9 mg
3	70.5 mg ± 0.5 mg F= 1.49 t = 17.39	69.9 mg ± 0.3 mg F = 3.31 (19*) t = 21.1 (2.77*)	67.2 mg ± 0.8 mg

a. Mean of three measurements;

* Tabulated value (n=3).

**Table 6.** Determination of lead in galena rock by using potentiometric sensors and atomic absorption spectrometry (AAS) techniques.

**Sample**	**Lead content^a^ (mg/g)**		
	**Sensor 3**	**Sensor 4**	**AAS [8]**
1	12.2 mg ± 0.8 mg	12.0 mg ± 0.5 mg	12.5 mg ± 0.9 mg
2	12.7 mg ± 0.4 mg	12.9 mg ± 0.2 mg	12.7 mg ± 0.8 mg
3	12.7 mg ± 0.8 mg F= 6.46 t = 0.222	12.1 mg ± 0.5 mg F = 18.69 (19*) t = 0.822 (2.77*)	12.4 mg ± 0.7 mg

a. Mean of three measurements;

* Tabulated value (n = 3).
